# A new *Tithaeus* species from Hainan Island, China (Arachnida, Opiliones, Laniatores, Epedanidae), with a key to the Chinese species

**DOI:** 10.3897/zookeys.67.705

**Published:** 2010-11-10

**Authors:** Chao Zhang, Feng Zhang

**Affiliations:** College of Life Sciences, Hebei University, Baoding Hebei 071002, China

**Keywords:** Opiliones, Laniatores, Tithaeus, new species, Hainan Island, China

## Abstract

A new species of the harvestmen Tithaeus calyptratus **sp. n.** (Epedanidae, Opiliones) from Hainan Island (China) is diagnosed, described and illustrated. A key to the two Chinese species of Tithaeus is provided.

## Introduction

**Figure 1.**
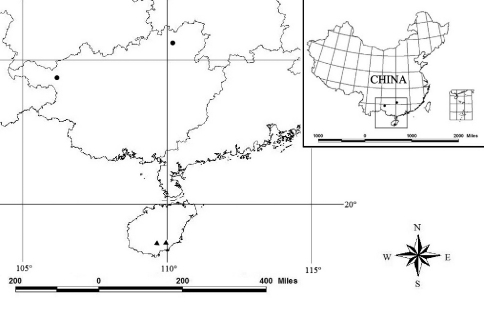
Distribution of Tithaenus drac (black circles) and Tithaenus calyptratus (black triangle) in China

The family Epedanidae Sørensen, 1886 is represented by 188 species in 73 genera worldwide (Kury 2003). The genus Tithaeus was described on the basis on the type species, Tithaenus laevigatus Thorell, 1891, from Malaysia. At present, the genus includes 34 valid species mainly distributed in South East Asia. Among them, the only species, Tithaeus drac Lian, Zhu & Kury, 2008, known from both sexes, has been reported from China (Fig. 1). However, the majority of the Tithaeus species remain poorly known, especially as far as their genital morphology concern. Therefore, the diversity of Tithaeus reported from south China (2 species) and neighbouring countries of SE Asia, such as Myanmar (1 species), Vietnam (1 species), Singapore (2 species), and Thailand (4 species), is much lower that that in Indonesia (7 species) or Malaysia (18 species) (Thorell 1891, Loman 1905, Roewer 1912, 1923, 1927, 1949, Banks 1930, Suzuki 1969a, 1969b, 1972, 1985, Lian et al. 2008).

During a 2009 faunal survey of tropical Hainan Island, a few specimens of the Laniatores were collected. Among them, one species of Tithaeus was identified as new to science and is described in this paper.

## Marterials and methods

Two males and one female were collected from Hainan Island in south China (Fig. 1). All type specimens are deposited in the Museum of the Hebei University (MHBU), Baoding, China. Specimens were preserved in 75% ethanol, examined and drawn using a Leica M165c stereomicroscope equipped with a drawing apparatus. The genitalia were first placed in hot lactic acid then moved to distilled water in order to expand them for observation (Schwendinger and Martens 2002). All measurements are in mm.

## Taxonomy

### Tithaenus

Thorell, 1891

Tithaeus Thorell 1891: 371; Banks 1930: 66; Lian et al., 2008: 53–54.
Sinis Loman 1892: 12.
Sinniculus Loman 1902: 198.

#### Type species:

Tithaeus laevigatus Thorell, 1891, by original designation.

#### Diagnosis and distribution:

see Lian et al. (2008).

#### Key to species of Tithaeus known in China.

**Table:** 

1.	Carapace flat between its anterior margin and ocularium	Tithaenus drac (Lian, Zhu & Kury, 2008)
–	Carapace with a low hump situated between its anterior margin and ocularium	Tithaenus calyptratus sp. n.

### Tithaeus calyptratus sp. n.

urn:lsid:zoobank.org:act:E8C66900-F2DD-4852-A753-4B54459832CB

Figs 2 17 

#### Type material.

The ♂ holotype (MHBU) from China, Hainan Province, Mt. Diaoluo [18.67° N, 109.92° E], 5 June 2009, C. Zhang leg.

Paratypes: 1♀ (MHBU), together with the holotype; 1♂ (MHBU), China, Hainan Province, Mt. Qixianling [18.77° N, 109.68° E], 9 June 2009, C. Zhang leg.

#### Etymology.

The specific name is derived from the Greek word “calyptra” meaning a cap or hat, referring to the straw-hat type stylar lobe of the penis.

#### Diagnosis.

The new species is similar to Tithaenus kokutnus Suzuki, 1985, recorded from northern Thailand (Suzuki, 1985: fig. 4), but can be easily distinguished from it by the following characters: (1) Cheliceral proximal segment armed with a large tooth and two smaller ones, situated medially on the ventral surface and the second segment is covered with granules on its frontal surface; (2) Both the dorsal margin of pedipalpal femur and its ventral margin between two setiferous tubercles are finely serrated; (3) Penis with a straw-hat shaped stylar lobe.

#### Comments.

Tithaenus calyptratus sp. n. has various morphological characters that support its placement in the genus Tithaeus: viz., scutal region with five areas, eye tubercle without a median spine, pedipalpus relatively short and thick, tarsi III and IV without scopulae and distitarsus I with two tarsalia. Furthermore, the genital characters (such as, the distal margin of the penial ventral plate with a deep cleft, glans with simple membranous lobe and each lobe of the ovipositor with two ventral and two dorsal setae) are also in agreement with to the generic disagnosis of Tithaeus (as per Lian et al. 2008).

#### Description.

**Figures 2–10.**
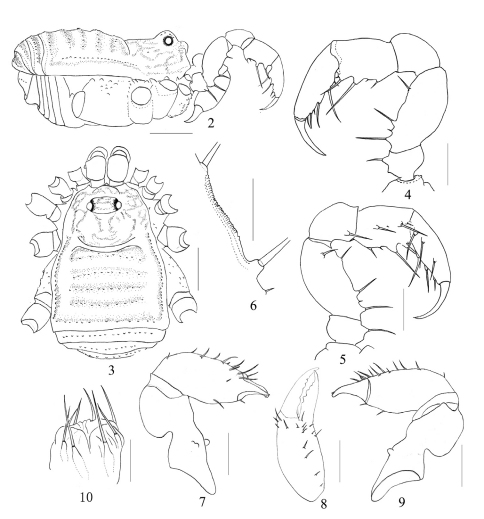
Tithaeus calyptratus sp. n. **2** Male body, lateral view **3** Same, dorsal view **4** Left pedipalpus, male, posterior view **5** Same, anterior view **6** Minute serrate margin on the ventral side of femur, left pedipalpus, male **7** Left chelicera male, anterior view **8** Distal segment of the left chelicera, male, above view **9** Left chelicera, male, posterior view **10** Ovipositor. Scale bars: 1mm (1–2); 0.5mm (3–4, 6–8); 0.25mm (5, 9).

Male (holotype) habitus as in Figs 2–3. Coloration: body rusty yellow; carapace and ocularium with yellow-brown reticulation; lateral margins and opisthosomal areas of scutum, and free tergites banded with blackish brown; all coxae and genital plate yellowish, free sternites somewhat clouded; chelicerae and pedipalpus yellowish, with brown reticulate markings above; trochanters of legs yellowish; femora to tarsi slightly darker.

Body from above as a trapezoid, wider posteriorly than anteriorly. Ocularium ovoid, only with a few granules. A low hump, lower than the ocularium, is situated between it and the anterior margin of carapace. Abdominal scutum, as well as each free tergite, with a transverse row of very small tubercles, and with a longitudinal row of granules on their lateral margins. Anal plate with scattered tubercles. Each of the free sternites with a row of obsolete granules. Coxa I with irregular hair-tipped granules, coxae II-IV smooth. Dorsal surface of coxa IV with several rather coarse granules. Coxa III with a few humps along the frontal and rear margins. Tracheal stigma clearly visible.

Chelicera (Figs 7–9). Proximal segment disto-dorsally visibly swollen, armed with a large tooth and two smaller ones, situated medially on the ventral surface. Second segment with some hair-tipped tubercles on frontal surface. Fingers relatively short but stout; inner edges toothed as shown in Fig. 8.

Pedipalpus (Figs 4–5) short and robust, trochanter with a ventral setaceous tubercle. Femur ventrally with three strong and a small setiferous tubercles; on the prolateral distal side with a setiferous tubercle. Femur dorsally with a minutely serrate margin (Fig. 6). Such a margin also between the two ventral setiferous tubercles. Patella disto-medially with a setiferous tubercle. Tibia ventro-laterally with two small and two prominent setiferous tubercles, ventro-medially with two stout and two reduced setiferous tubercles. Tarsus ventrally with three setiferous tubercles on each side.

Legs slender and relatively elongated. All segments unarmed, smooth. Femora I-IV straight. Tarsi III-IV with simple double claws, no scopulae. Tarsal formula: 5/11/5/6. Distitarsi of first and second tarsi each with two tarsalia.

**Figures 11–17.**
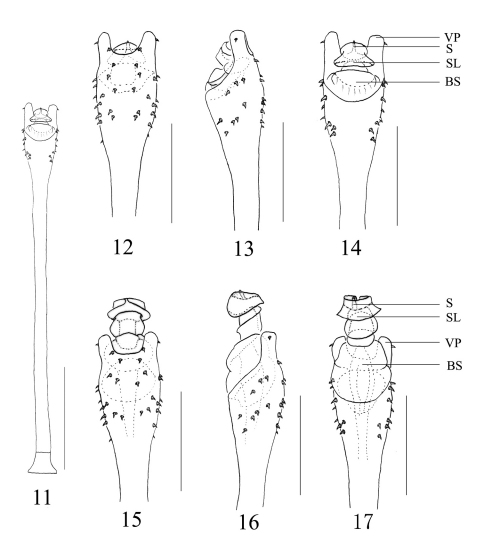
Tithaeus calyptratus sp. n. **11** Entire penis **12** Penis tip, ventral view **13** Ditto, lateral view **14** Ditto, dorsal view **15** Expanded penis, ventral view **16** Ditto, lateral view **17** Ditto, dorsal view. Abbreviations: **BS** basal sac **S** stylus **SL** stylar lobe **VP** ventral plate. Scale bars: 0.5 mm (10); 0.25mm (11–16).

Penis (11–17). Shaft slender and long, distal portion swollen. Ventral plate with a wide median cleft, setae arranged as shown in Figs 11–13. Basal sac oval, well developed, immovable and sunken into truncus. Glans with complex structures, twisted when at rest. Stylar lobe shaped somewhat like a straw hat and surrounding the stylus.

Female. Similar to male in general appearance but with a slightly larger body.

Ovipositor as illustrated (Fig. 10). Each lobe with two ventral and two dorsal setae.

Measurements: Male holotype (female paratype). Body 4.13 (4.44) long, 2.91 (3.06) wide at the widest portion, scutum 3.42 (3.52) long; eye tubercle 0.40 (0.38) long, 0.93 (0.85) wide. Pedipalpus claw 0.50 (0.63) long. Penis 1.78 long. Measurements of left pedipalpus and right legs as in Table 1.

**Table:** **Table 1.** Pedipalpus and leg measurements of the male holotype (female paratype).

	*Trochanter*	*Femur*	*Patella*	*Tibia*	*Metatarsus*	*Tarsus*	*Total*
Pedipalpus	0.35(0.38)	1.00(0.90)	0.70(0.68)	0.75(0.68)		1.00(0.90)	3.80(3.54)
Leg I	0.56(0.51)	2.70(2.35)	0.77(0.71)	2.19(1.89)	3.21(2.86)	1.68(1.73)	11.11(10.05)
Leg II	0.77(0.77)	6.32(5.30)	1.28(0.92)	5.97(4.74)	7.45(6.22)	4.28(4.34)	26.07(22.29)
Leg III	0.77(0.61)	3.57(2.91)	1.02(1.02)	2.40(2.09)	3.88(3.57)	2.04(2.04)	13.68(12.24)
Leg IV	0.71(0.71)	5.00(4.18)	1.12(1.02)	3.26(2.86)	5.61(5.20)	2.81(2.55)	18.51(16.52)

#### Habitat.

 Collected under fallen logs in the humid tropical forest.

#### Distribution.

 Hainan Province, China.

## Discussion

The opilionids genus Tithaeus was established by Thorell in 1891 (type species: Tithaenus laevigatus Thorell, 1891). Later, Roewer (1912, 1923, 1927, 1949) placed it in the subfamily Phalangodinae of Phalangodidae and Suzuki (1969a, 1969b, 1972, 1985) supported this assignment. However, recently Kury (2003, 2010) transferred Tithaeus to the Epedanidae. Lian et al. (2008) further considered the taxonomic status of the genus reasoning both from its somatic and from male genital morphology. Having compared Tithaeus similis Suzuki, 1985 with representatives of two subfamilies, the phalangodid and the epedanid, they found out that its male genitalia could be evidence of its relationship with the epedanid. We follow Kury’ opinion and consider Tithaeus a member of the Epedanidae on the basis of its genital characters, such as, a well developed immovable sac and the absence of complex introverting structures in the penis.

## Supplementary Material

XML Treatment for Tithaenus

XML Treatment for Tithaeus calyptratus
